# Phenotypic and genetic antimicrobial resistance of the intestinal microbiota isolated from two alpacas *(Vicugna pacos) post mortem*

**DOI:** 10.2478/jvetres-2025-0038

**Published:** 2025-09-30

**Authors:** Joanna Pławińska-Czarnak, Karolina Wódz, Zuzanna Janina Strzałkowska, Piotr Kwieciński, Monika Żychska, Ewa Dorota Domańska, Daria Kłosińska, Blanka Orłowska, Tomasz Nowak

**Affiliations:** 1 Department of Food Hygiene and Public Health Protection, Division of Veterinary Epidemiology and Economics, Division of Histology and Embryology, Department of Morphological Sciences, Institute of Veterinary Medicine, Warsaw University of Life Sciences – SGGW, 02-776 Warsaw, Poland; 2 Laboratory of Molecular Biology, Vet-Lab Brudzew, 62-720 Brudzew, Poland; 3 Division of Veterinary Epidemiology and Economics, Division of Histology and Embryology, Department of Morphological Sciences, Institute of Veterinary Medicine, Warsaw University of Life Sciences – SGGW, 02-776 Warsaw, Poland; 4 Division of Histology and Embryology, Department of Morphological Sciences, Institute of Veterinary Medicine, Warsaw University of Life Sciences – SGGW, 02-776 Warsaw, Poland

**Keywords:** antimicrobial resistance, intestinal microbiota, opportunistic pathogens, *Vicugna pacos*

## Abstract

**Introduction:**

In Poland, alpacas are commonly companion animals and producers of wool. Human-alpaca-environment interactions raise One Health concerns about antimicrobial resistance (AMR). No medications are licensed in Poland for camelids, and so all are prescribed under the cascade; they include β-lactams, cephalosporin, florfenicol, enrofloxacin, marbofloxacin, gentamicin, tetracycline and trimethoprim/sulfamethoxazole. Human and animal bacterial AMR is a matter of global concern. Consequently, the aim of the present study was to determine the prevalence of phenotypic and genotypic AMR among bacteria isolated from alpaca intestines.

**Material and Methods:**

Fifty-four strains were identified using matrix-assisted laser desorption/ionisation–time-of-flight mass spectrometry and biochemical methods. Antibacterial susceptibility was assessed by determining minimum inhibitory concentrations and by the Kirby–Bauer method.

**Results:**

*Citrobacter* spp., *Enterobacter* spp. and *Serratia* spp. exhibited resistance to β-lactams, first-generation cephalosporins and tetracyclines, with *Serratia* spp. also resistant to colistin, polymyxin B and florfenicol. *Enterococcus* spp. were resistant to penicillin G, benzylpenicillin and erythromycin, but not to vancomycin, while *Staphylococcus* spp. showed resistance to amoxicillin and penicillins, but not to methicillin. *Bacillus* spp. and *Corynebacterium* spp. were resistant to some penicillins, tetracyclines and trimethoprim-sulfamethoxazole. *Enterobacteriaceae* isolates carried resistance genes (*aadA, dfrA1, tetA, sul1, sul2, strA/strB* and *floR*); therefore, the tested alpacas’ microbiomes harboured AMR determinants.

**Conclusion:**

Alpacas should be monitored over an extended period to know the risk of transmission of AMR genes from components of their microbiome.

## Introduction

The alpaca (*Vicugna pacos*) is a domesticated species of camelid native to the Andean region. In Peru, alpacas possess important cultural value due to their historical significance and generate substantial economic benefits for communities involved in alpaca breeding by providing high-value meat with low cholesterol content, as well as skin and hypoallergenic fibre. In Poland, these animals are companion animals in petting zoos, or are used for therapeutic purposes and for wool production.

It is evident that gut-associated microbes have a significant impact on the nutritional status and health of their hosts, particularly where these hosts consume diets high in cellulose, as is the case with alpacas; unfortunately, little is known of their intestinal microbial population. There is one publication in which the effect of diet was investigated on the microbiota present in the forestomach compartment and the small and the large intestine of alpacas (5). Being frequent companion animals, alpacas are often in close contact with humans, and there is a need to enhance our understanding of the bacteria that constitute their gut microbiota and the potential drug resistance of the bacteria therein. The trend towards increasing drug resistance noted among microorganisms such as methicillin-resistant *Staphylococcus aureus* (MRSA) and totally drug-resistant and multidrug resistant *Mycobacterium tuberculosis* strains is an alarming one that demands urgent attention. In recent times, there have been concerted efforts to minimise the use of antibiotics in veterinary medicine, particularly with regard to farm animals, with a view to curbing the emergence of drug-resistant variants of pathogenic microorganisms. Drug resistance in environmental pathogens is a threat, it is essential to monitor continuously in application of the One Health principle, which is also the rationale for investigating innovative treatment options (17, 24, 28, 31, 32).

Bacteria have evolved various mechanisms for acquiring antimicrobial resistance (AMR), including the exchange and implementation of antimicrobial resistance genes. These resistance genes are frequently found on plasmids or transposons, which can be transferred between cells *via* conjugation, transformation or transduction. Bacterial communities (*i.e*. microbiota) rapidly acquire antibiotic resistance through such gene transfer, and this resistance spreads within and between different bacterial species inhabiting the same environment, such as the intestines (3, 20). One factor believed to have a significant impact on the transmission of antibiotic-resistant strains is the acquisition of antibiotic resistance by components of the microbiota of wild animals. It is conceivable that wild animals may act as carriers for these antibiotic-resistant strains, thereby contributing to the dissemination of resistance genes in the environment. From the soil and water environmental compartments, resistant strains may be transferred to other ecosystem members through contact.

The potential for the transmission of antibioticresistant microbes between humans and companion or wild animals is therefore a matter of concern. There is a need for greater monitoring of drug resistance in both pathogenic bacteria and in the natural microbiota and the environment, as this is a crucial step in identifying potential threats to both human and animal health (2, 18). Fortunately, recent years have seen a marked increase in our understanding of the role played by the bacterial microbiota in many animal species and in our awareness of microbiome elements’ potential to be shared among animals, humans and the environment. While alpacas are considered to be relatively disease resistant, they are believed to be at risk of parasitic infestations and diarrhoea (2).

Amikacin, ampicillin, ceftiofur, amoxicillin, amoxicillin and clavulanic acid, florfenicol, enrofloxacin, marbofloxacin, gentamicin, tetracycline, tobramycin and trimethoprim/sulfamethoxazole are used in alpaca treatment (15, 16). These drugs increasingly often fail against phenotypically and genotypically resistant bacteria, and it is strategically important to learn if strains of such bacteria are prevalent in particular species of farmed animals. Given that in Poland, the past two decades have demonstrated a growing trend in the breeding of newly introduced animal species including alpacas, which were officially recognised as farm animals in 2021, this study was planned to determine the phenotypic and genotypic drug resistance of bacteria isolated *post mortem* from the intestines of two alpacas with diarrhoea.

## Material and Methods

### Sampling

Swabs (sterile Amies; Deltalab, Rubí, Spain) were taken from the small intestine, colon, rectum and faeces of two dead alpacas (*Vicugna pacos*) during their dissection. The procedure was performed in the Department of Veterinary Pathology and Diagnostics, Institute of Veterinary Medicine, Warsaw University of Life Sciences in Poland.

The first alpaca (1) was a male approximately 10 months old, which had been brought to the veterinary clinic because it had been badly neglected. The animal was in very poor health, suffering from diarrhoea and severe lesions due to mange infestation, which covered almost its entire body. The alpaca died during transport to the clinic.

The second alpaca (2) was a 12-month-old female that came from a herd that had not received any veterinary care, and was also brought to the veterinarian because it had been neglected. The animal was in poor health, with diarrhoea and severe, extensive mange lesions over almost all of its skin. The animal experienced sudden weakness and also died during transport. A more detailed description of the animals is provided by Pławińska-Czarnak *et al*. (23).

### Bacteria isolation and identification

Isolation was carried out with the use of basic microbiological media prepared according to the manufacturer’s instructions: Baird-Parker (BTL, Lodź, Poland) and Oxoid chromogenic listeria agar (Oxoid, Basingstoke, UK), *Yersinia* cephsulodin, irgasan and novobiocin agar (a selective medium for the isolation of *Yersinia* spp.), Columbia agar with 5% sheep’s blood, modified charcoal cephoperazone deoxycholate blood-free selective *Campylobacter* agar, mannitol salt agar (Chapman), mannitol egg yolk polymyxin agar, Columbia colistin and nalidixic acid agar with 5% sheep blood and MacConkey’s agar (all from Graso, Gdańsk, Poland). The incubation conditions were in line with the manufacturers’ recommendations. Briefly, 24-h nutrient agar or Columbia agar cultures with 5% sheep blood were used to identify bacteria by matrix-assisted laser desorption/ionisation–time-of-flight mass spectrometry (MALDI-TOF MS), the automated VITEK2 and analytical profile index (API) 20E, API 20NE and API Staph card biochemical test systems (bioMérieux, Marcy-l’Étoile, France), and rapID One and rapID NF Plus assays (Thermo Fisher Scientific, Lenexa, KN, USA).

For the phenotypic assessment of antibiotic resistance, 54 bacterial strains found in alpacas were selected. These included the following Gram-negative bacteria: *Citrobacter freundii, C. braakii, Enterobacter cloacae, Ent. gergoviae, Ent. hormaechei, Ent. ludwigii* and *E. coli*. The following members of the *Yersiniaceae* family were selected: *Serratia liquefaciens, Ser. odorifera* and *Ser. marcescens*. The Gram-positive cocci included *Enterococcus hirae, Entc. gallinarum, Entc. faecium, Entc. casseliflavus, S. aureus, S. sciuri* and *Micrococcus luteus. Bacillus altitudinis, B. licheniformis, B. cereus, B. cecembensis, B. flexus, B. pumilus, B. subtilis* and *B. aeolius* were the aerospore-forming bacilli assessed. The remainder of the bacterial isolates tested for resistance were *Moraxella osloensis, Leuconostoc citreum, Corynebacterium diphtheriae* and *Cor. stationis*.

### Antimicrobial sensitivity testing

The antimicrobial susceptibility of the 54 tested bacterial strains was assessed by determining the minimum inhibitory concentrations (MICs) using a 96-well MICRONAUT Special Plate with the following antimicrobials: amoxicillin and clavulanic acid (AMC), amoxicillin (AMO), cefquinome (CEQ), ceftiofur (CTF), cephalexin, cloxacillin, colistin (COL), cephapirin, doxycycline (DOX), enrofloxacin (ENR), erythromycin (ERY), florfenicol (FLO), gentamicin (GEN), lincomycin, lincomycin/spectinomycin, nafcillin, neomycin (NEO), norfloxacin (NOR), oxytetracycline (OXY), penicillin G (PG), streptomycin (STR), trimethoprim-sulfamethoxazole (SXT), tiamulin, tylosin (TYL) and tylvalosin (Merlin Diagnostika, Bornheim, Germany).

In addition, antimicrobial susceptibility tests (AST) were conducted using the automated VITEK 2 System, AST-GN96 cards for Gram-negative bacteria and AST-GP79 cards for Gram-positive bacteria (bioMérieux). The AST card is essentially a miniaturised and streamlined version of the doubling dilution technique for MICs determined by the microdilution method. The AST-GN96 card was used to determine susceptibility to ampicillin (AMP), AMC, CLE, cephalotin (CF), cephoperazone, CTF, CEQ, imipenem (IMI), GEN, NEO, flumequine (UB), ENR, marbofloxacin (MAR), polymyxin B (PB), tetracycline (TET), FLO, SXT and ticarcillin/clavulanic acid. Using AST-GP79, the antimicrobial susceptibility was assessed of *Enterococcus* and *Staphylococcus* to benzylpenicillin (BEN), AMP, oxacillin, CF, CTF, CEQ, amikacin, GEN, NEO, ENR, ERY, tilmicosin, clindamycin (CLI), TET, FLO, kanamycin, SXT and TYL. The EUCAST 2023 breakpoints were applied in evaluation of the resistance of all bacterial isolates (11). The Clinical and Laboratory Standards Institute (CLSI) M45 method standard breakpoints (8) were referred to in the course of resistance testing of all isolates except those of the *Bacillus, Moraxella, Corynebacterium* and *Leuconostoc* genera. The resistance of isolates of these four genera was assessed making relevant use of the CLSI M100 performance standard (10). These same four genera and *Stenotrophomonas* were also cross-referenced for their resistance against CLSI VET06 breakpoints (9), whereas the VET01 interpretive criteria (7) were applied to all other isolates.

The obtained MIC values were subjected to quality control using *E. coli* American type culture collection (ATCC) 25922, *S. aureus* ATCC 29213 and *Entc. faecalis* ATCC 29212.

All the *Enterococcus* and *Leuconostoc citreum* isolates were additionally tested for antibiotic sensitivity against vancomycin (VAN; 30 μg) and teicoplanin (TEI; 30 μg) by the Kirby–Bauer disc-diffusion method using commercially available discs (Oxoid) and Muller– Hinton agar medium (Graso). The disc-diffusion test readings were taken at 24 h. Quality control of the antibiotic discs was carried out using *Entc. faecalis* ATCC 29212 as a VAN-sensitive strain and *Entc. faecalis* ATCC 49533 as a strain susceptible to GEN, VAN and TEI and resistant to STR.

The *Staphylococcus* isolates were also tested by cefoxitin disc diffusion on Mueller–Hinton agar using a 30 μg cefoxitin disc. The plates were incubated at 35°C for 18 h and the zone diameters were measured thereafter.

### Bacterial DNA preparation and conventional single and multiplex polymerase chain reaction

Bacterial DNA for PCR tests was extracted using the commercial DNA Extraction Mix II (Kylt, Höltinghausen, Germany). The *aadA, strA/strB, aphA1, aphA2, aadB, tetA, tetB, sul1, sul2, sul3, dfrA1, dfrA10, dfrA12, floR, bla*TEM, *bla*SHV, *bla*CMY-2, *bla*PSE-1 and *bla*CTX-M antimicrobial resistance genes were detected by conventional PCR, using specific primer pairs in multiplex or a single reaction. The primer sequences, predicted PCR product sizes, annealing temperature and references were previously described by Pławińska-Czarnak *et al*. (22).

### Statistical Assessment

Statistical tests were performed using Statistica software, version 13.1 (TIBCO, Palo Alto, CA, USA). Descriptive statistics were calculated to determine the proportions of isolates resistant to antimicrobial agents. The significance of any differences between proportions was determined by chi-squared tests.

## Results

All bacteria were identified and characterised as described in a previous publication by the authors (23). Most of the bacteria isolated were Gram-positive, including dominant species such as *Bacillus* spp., *Enterococcus* spp. and *Staphylococcus* spp. Other Gram-positive bacteria identified included *Cor. diphtheriae* and *Cor. stationis. Micrococcus luteus* was also isolated, being variously indicated as Gram-positive or Gram-variable. Various Gram-negative bacteria were also identified, including *Ent. cloacae, Ent. hormaechei, Ent. ludwigii* and *Ent. gergoviae*; *Citrobacter* spp.; *E. coli*; and *Stenotrophomonas maltophilia, Ser. liquefaciens, Ser. odorifera* and *Ser. marcescens*. No resistance against IMI or COL was detected when all isolates of *Citrobacter* and *Enterobacter* (confirmed to be so by MALDI-TOF MS and biochemical tests) were subjected to microdilution MIC testing using the MICRONAUT plates as described above.

All *C. freundii* were resistant to AMP, AMO, AMC, first-generation cephalosporins, tetracyclines, FLO and SXT. *Citrobacter freundii* isolated from alpaca 1 was resistant to fluoroquinolones (ENR and NOR), while those isolated from the faeces of alpaca 2 were only resistant to NOR. All tested strains were resistant to second-generation fluoroquinolones (ENR and NOR), but not to the third-generation MAR. All *Enterobacter* strains were resistant to AMP and first generation cephalosporins, seven *Enterobacter* strains were additionally resistant to AMO, three were to AMC and four were resistant to SXT. *Escherichia coli* was resistant to AMP, AMO and CF (Table 1). Judged against the TECOFF, *E. coli* showed resistance to STR (MIC > 16) (Supplementary Tables S1, S2 and S3). No IMI resistance was detected in any isolated species. No extended-spectrum β-lactamases (ESβLs) were detected by the screening in AST-GN96.

**Table 1.** Antimicrobial resistance pattern of Gram-negative *Enterobacteriaceae* isolates from two alpacas’ intestinal swabs and faeces

*Enterobacteriaceae* rods	Sample source (animal)	Antimicrobial resistance profile	Antimicrobial resistance gene
*Citrobacter freundii*	rectum (1)	AMP-AMO-AMC-CLE-CF-DOX-TET-FLO-SXT-ENR-NOR	*tetA, sul1, floR*
*Citrobacter freundii*	rectum (1)	AMP-AMO-AMC-CLE-CF-DOX-TET-FLO-SXT-ENR-NOR	*tetA, sul1, floR*
*Citrobacter freundii*	faeces (1)	AMP-AMO-AMC-CLE-CF-DOX-TET-FLO-SXT-ENR-NOR	*tetA, sul1, floR*
*Citrobacter freundii*	faeces (2)	AMP-AMO-AMC-CLE-CF-DOX-TET-FLO-SXT-NOR	*aadA, tetA, sul1*
*Citrobacter braakii*	small intestine (1)	DOX-TET-FLO-SXT	*floR*
*Enterobacter cloacae*	colon (1)	AMP-AMO-CF-FLO-SXT	*sul1, sul2, floR*
*Enterobacter cloacae*	colon (1)	AMP-AMO-CF-FLO-SXT	*sul1, sul2, floR*
*Enterobacter cloacae*	colon (1)	AMO-CF-DOX-TET-ENR-NOR	*tetA*
*Enterobacter cloacae*	rectum (2)	AMP-AMO-CF-ENR-NOR	-
*Enterobacter cloacae*	faeces (2)	AMP-AMO-AMC-CLE-CF-SXT	*sul1*
*Enterobacter gergoviae*	small intestine (2)	AMP-AMO-AMC-CLE-FLO	-
*Enterobacter hormaechei*	colon (2)	AMP-AMO-CLE-CF-FLO-SXT	*sul1, dfrA1*
*Enterobacter ludwigii*	rectum (1)	AMP-AMO-AMC-CLE-CF-FLO	-
*E. coli*	colon (1)	AMP-AMO-AMC-CF-STR	*aadA, strA/strB*

1 AMP – ampicillin; AMO – amoxicillin; AMC – amoxicillin/clavulanic acid; CLE – cephalexin; CF – cephalotin; DOX – doxycycline; TET – tetracycline; FLO – florfenicol; SXT – trimethoprim/sulfamethoxazole; ENR – enrofloxacin; NOR – norfloxacin; STR – streptomycin

**Table 2.** Antimicrobial resistance pattern of Gram-negative *Yersiniaceae* isolates from two alpacas’ intestinal swabs and faeces

*Yersiniaceae* rods	Sample source (animal)	Antimicrobial resistance profile	Antimicrobial resistance gene
*Serratia liquefaciens*	colon (2)	AMO-AMC-CLE-CF-COL-PB-DOX-TET-FLO	*tetA*
*Serratia liquefaciens*	small intestine (2)	CLE-CF-COL-PB-FLO	-
*Serratia odorifera*	faeces (2)	AMO-AMC-CLE-CF-COL-PB-FLO	-
*Serratia odorifera*	rectum (2)	CLE-CF-COL-PB-FLO	-
*Serratia marcescens*	faeces (1)	AMO-AMC-CF-COL-PB-DOX-TET-FLO	*tetB*

1 AMO – amoxicillin; AMC – amoxicillin/clavulanic acid; CLE – cefalexin; CF – cephalotin; COL – colistin; PB – polymyxin B; DOX – doxycycline; TET – tetracycline; FLO – florfenicol

The *TetA* gene, encoding the tetracycline efflux pump, was found in *C. freundii, Ent. cloacae* and *Ser. liquefaciens*; all species exhibited phenotypic resistance to tetracyclines. In addition, *Ser. marcescens* was found to harbour *tetB*, encoding a tetracycline efflux protein which confers resistance to TET and DOX. Both the *sul1* and *sul2* genes, associated with sulphonamide resistance, were found in *Ent. cloacae* isolated from the colon swab of alpaca 1. In addition, *sul1* was detected in all *C. freundii*, in *Ent. cloacae* isolated from the colon of alpaca 1 and the faeces of alpaca 2, and in SXT-resistant *Ent. hormaechei*. Interestingly, no *tet* or *sul* genes were detected in *C. braakii*, despite it demonstrating phenotypic resistance to tetracyclines and SXT. The *aadA* gene, encoding resistance to STR, was detected in sSTR-susceptible *C. freundii* and STR-resistant *E. coli*, together with the STR resistance genes *strA* and *strB*. All *Enterococcus* species were resistant to PG, BEN and ERY. *Entc. gallinarum, Entc. faecium* and *Entc. hirae* were resistant to aminoglycosides (GEN, NEO and STR), but *Entc. casseliflavus* was not. *Enterococcus casseliflavus, Entc. gallinarum* and *Entc. hirae* were resistant to ERY, but *Entc. faecium* was not. Four strains were TET resistant, and *Entc. hirae* and *Entc. casseliflavus* isolated from the colon and rectum of alpaca 2 were resistant to FLO. The analysis based on TECOFFs indicted that *Entc. faecium* showed resistance to GEN (MIC > 32 μg/mL), NEO (MIC > 64 μg/mL) and STR (MIC > 128 μg/mL). *Enterococcus hirae* showed resistance to GEN (MIC > 32 μg/mL), NEO (MIC > 128 μg/mL) and STR (MIC > 128 μg/mL). No resistance to vancomycin or teicoplanin was detected in any *Enterococcus* strains.

Four *Staphylococcus* species were resistant to AMO. *Staphylococcus pseudintermedius* and *S. sciuri* were resistant to PG and BEN, but *S. aureus* was not. Two *S. aureus* strains were resistant to ENR, and *S. pseudintermedius* was resistant to ERY. None of the *Staphylococcus* strains demonstrated methicillin resistance, as indicated by cefoxitin disc diffusion or AST-GP79 cards. *Micrococcus luteus* was resistant to PG, BEN, ERY and CLI. When cross-referenced with the TECOFF, *S. aureus* demonstrated resistance to NEO (MIC > 1 μg/mL) and STR (MIC > 16 μg/mL) and *S. pseudintermedius* to TYL (MIC >2 μg/mL). *Micrococcus luteus* was resistant to PG, BEN, ERY and CLI according to the CLSI M45 method standard (8) (Table 3).

**Table. 3.** Antimicrobial resistance pattern of Gram-positive cocci isolates from two alpacas’ intestinal swabs and faeces

Gram-positive cocci species	Sample source (animal)	Antimicrobial resistance profile	(T)ECOFF*
*Enterococcus faecium*	small intestine (1)	PG-BEN-NOR-DOX-TET	GEN-NEO-STR
*Enterococcus hirae*	small intestine (1)	PG-BEN-NOR-ERY	GEN-NEO-STR
*Enterococcus hirae*	colon (2)	PG-BEN-NOR-ERY-DOX-TET-FLO	GEN-NEO-STR
*Enterococcus hirae*	rectum (2)	PG-BEN-NOR-ERY-DOX-TET-FLO	GEN-NEO-STR
*Enterococcus casseliflavus*	colon (2)	PG-BEN-ERY-FLO	-
*Enterococcus casseliflavus*	rectum (2)	PG-BEN-ERY-FLO	-
*Enterococcus gallinarum*	rectum (1)	PG-BEN-DOX-TET-NOR-ERY	GEN-NEO-STR
*Staphylococcus aureus*	colon (1)	AMO-ENR	NEO-STR
*Staphylococcus aureus*	rectum (1)	AMO-ENR	NEO-STR
*Staphylococcus pseudintermedius*	rectum (2)	AMO-PG-BEN-ERY	TYL
*Staphylococcus sciuri*	colon (1)	AMO-PG-BEN	-
*Staphylococcus sciuri*	small intestine (2)	PG-BEN	-
*Micrococcus luteus*	rectum (2)	PG-BEN-ERY-CLI	-

1 PG – penicillin G; BEN – benzylpenicillin; NOR – norfloxacin; DOX– doxycycline; TET – tetracycline; GEN – gentamicin; NEO – neomycin; STR – streptomycin; ERY – erythromycin; FLO – florfenicol; NOR – norfloxacin; AMO – amoxicillin; ENR – enrofloxacin; TYL – tylosin; CLI – clindamycin;

* – tentative epidemiological cut-off value from European Committee on Antimicrobial Susceptibility Testing data

All *Bacillus* species were resistant to PG and SXT. Thirteen of them were resistant to ERY, three were resistant to tetracyclines (DOX and OXY) and one (*B. flexus*) to GEN (Table 4, Supplemenatary Tables S4 and S5).

**Table 4.** Antimicrobial resistance pattern of *Bacillus* spp. isolates from alpaca 2’s intestinal swabs and faeces

*Bacillus* species	Sample source (animal)	Antimicrobial resistance profile
*Bacillus altitudinis*	small intestine	PG-ERY-SXT
*Bacillus licheniformis*	small intestine	PG-DOX-OXY-ERY-SXT
*Bacillus licheniformis*	small intestine	PG-SXT
*Bacillus cereus*	colon	PG-ERY-SXT
*Bacillus cereus*	colon	PG-ERY-SXT
*Bacillus cecembensis*	colon	PG-SXT
*Bacillus flexus*	colon	PG-ERY-SXT
*Bacillus licheniformis*	colon	PG-GEN-SXT
*Bacillus licheniformis*	colon	PG-ERY-SXT
*Bacillus pumilus*	colon	PG-ERY-SXT
*Bacillus pumilus*	colon	PG-ERY-SXT
*Bacillus pumilus*	colon	PG-ERY-SXT
*Bacillus subtilis*	colon	PG-ERY-SXT
*Bacillus subtilis*	colon	PG-ERY-SXT
*Bacillus licheniformis*	rectum	PG-DOX-OXY-ERY-SXT
*Bacillus aeolinus*	rectum	PG-DOX-OXY-ERY-SXT
*Bacillus cereus*	rectum	PG-SXT

1 PG – penicillin G; ERY – erythromycin; SXT – trimethoprim/sulfamethoxazole; DOX – doxycycline; OXY – oxytetracycline

The antimicrobial resistance profiles of the remaining isolates were also analysed. The anatomical source of resistant strains and the corresponding profiles are presented in Table 5.

**Table 5.** Antimicrobial resistance pattern of miscellaneous isolates from two alpacas’ intestinal swabs and faeces

Species	Sample source (animal)	Antimicrobial resistance profile
*Moraxella osloensis*	small intestine (2)	PG-ERY
*Leuconostoc citreum*	rectum (2)	PG
*Corynebacterium diphtheriae*	colon (1)	PG-DOX-OXY-ERY-SXT
*Corynebacterium stationis*	colon (1)	PG-DOX-OXY-ERY-SXT
*Stenotrophomonas maltophilia*	colon (1)	DOX-OXY-SXT

Both *Cor. diphtheriae* and *Cor. stationis* were resistant to penicillin, tetracyclines (DOX and OXY), ERY and SXT. *Leuconostoc citreum* was resistant only to PG and *Stenotrophomonas maltophilia* to SXT. (Supplementary Tables S6, S7, S8, S9, S10, S11, and S12).

## Discussion

Few works have examined the microorganisms in the microbiota of New World camelids (the guanaco, vicuña, llama and alpaca) (25). As these animals may have the potential to transmit zoonotic infections, it is crucial that veterinarians possess a comprehensive understanding of the bacterial infections suffered by alpacas, the species with which humans interact most. This need is even more pressing as alpaca breeding has recently gained popularity in regions where they are not indigenous, including Poland. Close interaction may also result in increased transmission of bacteria between humans and alpacas. Even if non-pathogenic bacteria are transferred between humans and animals, each new colonisation has the potential to modify the composition of the gastrointestinal microbiome, which may impair the ability of the host to resist colonisation by exogenous bacteria. If pathogenic and antibiotic-resistant strains are transferred, each cross-infection is a part of the global spread of AMR, which has been recognised as a matter of significant concern and a motive for continuous epidemiological surveillance in both human and nonhuman hosts.

The present study examined the intestinal bacterial composition of alpacas to evaluate the antibioticresistant profiles at both the genotypic and phenotypic level. Our findings confirmed the presence of antibioticresistant bacteria in animals that had not previously been exposed to antibiotics. Genes for AMR were identified in *C. braakii, C. freundii, Ent. cloacae, Ent. hormaechei* and *E. coli*. The identified bacteria also demonstrated varied resistance profiles, *Citrobacter, Serratia* and *Enterococcus* spp. exhibiting the highest level of resistance as determined by CLSI and EUCAST breakpoints.

It is possible that *Enterobacterales* may demonstrate resistance to first-line antibiotics such as ENR, GEN, OXY, penicillin, or SXT, because these antibiotics have been commonly used for many years by veterinarians in treating infections with bacteria in this taxonomic order in cattle, chickens and pigs. Enrofloxacin is used in the treatment of a wide range of farm and exotic animals because of its safety and efficacy against a variety of pathogens; however, our findings indicate that three *C. freundii* and two *Ent. cloacae* isolates were resistant to this antibiotic. All *Enterobacter* species are intrinsically resistant to AMP, AMO and first-generation cephalosporins owing to their production of constitutive AmpC, a chromosomal group 1 β-lactamase.

Our findings also indicate that all the *Citrobacter* isolates were resistant to FLO; however, while four of the five isolates carried the *floR* gene, it was not present in the *C. freundii* isolated from the faeces of alpaca 2. The same was noted for the *Serratia* strains. The bacteria positive for the *floR* gene had much higher MICs for FLO than the *floR*-negative strains (33). *Escherichia coli* isolated from the colon (1) was resistant to AMP, AMO, CF and STR. The *aadA* and *strA/strB* genes encode resistance to STR, and were detected in STR-resistant *E. coli*. No ESβL-producing strains were found in this study, unlike in the study of Cardenas-Arias *et al*. (4), which reported the presence of ESβL-producing *E. coli* ST10 in llamas.

*Enterococcus hirae wa*s the most frequently-detected species, followed by *Entc. casseliflavus, Entc. gallinarum* and *Entc. faecium*. This is consistent with data obtained for other camelids (14). It is well known that *Enterococcus* spp. exhibit decreased susceptibility to penicillin and AMP, as well as to semisynthetic penicillins, because they express low-affinity penicillin-binding proteins. Our findings also identified resistance to aminoglycosides (GEN, NEO and STR), ERY and FLO; a similar profile of dissemination of antibiotic resistance was previously noted for *Enterococcus* spp. isolated from camelids (14). In contrast, *Bacillus* and *Staphylococcus* spp. exhibited the highest susceptibility to all tested antibiotics.

The findings of the present study on *Staphylococcus* are consistent with those reported in previous research on camelids (21). Nonetheless, several significant differences were noted, particularly in relation to the occurrence of drug resistance. Staphylococci are regarded as opportunistic pathogens, manifesting as colonisers and demonstrating the capacity to induce a broad spectrum of severe infections. *Staphylococcus sciuri* is often detected in animals, humans and the environment, where it is regarded as non-pathogenic; it has also been identified in diseased animals (21, 26). In our study, staphylococci showed resistance to AMO, PG, BEN, ENR and ERY. The TECOFFs imply that *S. aureus* resists NEO and STR, and that *S. pseudintermedius* resists TYL. Methicillin-resistant *S. aureus* has been identified as one of the most significant multidrug-resistant pathogens globally. The prevalence of MRSA among New World camelids is low but nevertheless alarming (26). In the current study, the tested *Staphylococcus* strains did not demonstrate methicillin resistance in cefoxitin disc diffusion or screening in AST-GP79 cards. However, previous research has identified β-lactam resistance in llamas and alpacas (27). *Staphylococcus sciuri* isolated from alpacas was found to display multiple resistance, with the bacteria being non-susceptible to at least one antimicrobial agent from three or more antibiotic classes (21).

*Bacillus* strains isolated from alpacas were susceptible to most tested antibiotics, but all *Bacillus* species were resistant to PG and SXT. Thirteen of the seventeen *Bacillus* isolates were resistant to ERY, and three to tetracyclines. *Bacillus flexus* was the only species resistant to GEN, which is consistent with the findings of another study (12).

*Moraxella osloensis* isolated from the small intestine of alpaca 2 showed resistance to PG and ERY. The prevalence of macrolide-resistant *Moraxella* has given rise to concerns regarding therapeutic interventions in cases of infection with species in this genus, particularly in children suffering from pneumonia caused by *M. catarrhalis*. Macrolide-sensitive *Moraxella* cells rapidly accumulate large amounts of macrolide antibiotics, whereas no such accumulation was observed in macrolide-resistant cells with smooth-type or rough chemotype c lipopolysaccharide (30).

*Stenotrophomonas maltophilia* has been identified as an opportunistic pathogen of significant concern to susceptible humans. It is known to play an important role as a nosocomial pathogen associated with infections, especially in immunocompromised or debilitated patients, and demonstrates remarkable intrinsic AMR and acquired resistance to multiple antimicrobial agents. In the present study, the *Stenotrophomonas maltophilia* isolated from the colon of alpaca 1 was found to be resistant to tetracyclines and SXT. However, the lack of reproducible AST, lack of clinical breakpoints and an absence of clinical outcome data rule out regarding these results as conclusive.

The *Micrococcus luteus* and *Leuconostoc citreum* isolated from the alpaca gut microbiota were different from those adapted to humans, and they remained free of antibiotic resistance mechanisms. *Micrococcus luteus* was resistant to PG, BEN and ERY, and one isolate was to CLI, while *Leuconostoc citreum* was only resistant to PG. Interestingly, while *Corynebacterium* spp. were resistant to penicillin, the tetracyclines, ERY and SXT in the present study, no such resistance was observed for *Cor. pseudotuberculosis* isolated from alpacas afflicted with lymphadenitis or subcutaneous abscesses by Anderson *et al*. (1). All strains were susceptible to tetracyclines, ERY and SXT.

A few studies have described the microbiota of alpacas and related camelids, including those of the eye (13, 29), ear (19), and skin (6). However, only a small number of studies have focused on the gut microbiota. Research into the microorganisms present in exotic animals such as alpacas and the antibacterial resistance profiles of those microorganisms is of critical importance, particularly in view of the dissemination of these bacteria both within the environment and between humans and other animal species.

## Conclusion

Our findings indicate that post-mortem sampled bacterial strains constituting the intestinal microbiota of alpacas had different antibiotic resistance profiles at the genotypic and phenotypic levels. Many of the bacteria isolated from the intestinal tracts of the alpacas demonstrated high antibiotic susceptibility, which is most likely due to low direct exposure to antibiotics. This absence of resistance could suggest that the capacity for transmission of antimicrobial resistance from alpacas to humans is limited. However, while most bacteria isolated from alpacas were different from those adapted to humans, our results indicated that potentially pathogenic isolates were also present in the alpaca intestine. Therefore, to confirm the risk of zoonoses and the potential transmission of AMR genes, it is recommended that monitoring should be extended over a longer period. As a response to the increase in the popularity of alpacas for recreation in recent years, more research on New World camelids should be carried out, especially when considering their increasing proximity to domestic and domiciled animals, as well as humans.

## Supplementary Material

Supplementary Material Details
