# Supplementary material for: Phenotypic and genetic antimicrobial resistance of the intestinal microbiota isolated from two alpacas (Vicugna pacos) post mortem
Source: J Vet Res. 2025 Sep 30;69(3):345–52. doi: 10.2478/jvetres-2025-0038 (PMC12503216; doi:10.2478/jvetres-2025-0038)
Supplement: Supplementary file 1 — Supplementary Material Details [file jvetres-2024-038_sm.pdf]

Supplementary Table S1. Clinical and Laboratory Standards Institute M100 Ed31 Performance Standards for Antimicrobial Susceptibility Testing breakpoints for *Enterobacterales*

Antimicrobial	Minimum inhibitory concentration (µg/mL)		
	Susceptible	Intermediate	Resistant
Ampicillin	≤8	16	≥32
Amoxicillin/clavulanic acid	≤8/4	16/8	≥32/16
Doxycycline	≤4	8	≥16
Gentamicin	≤4	8	≥16
Norfloxacin	≤4	8	≥16
Tetracycline	≤4	8	≥16
Trimethoprim/sulfamethoxazole	≤2/38	-	≥4/76

Supplementary Table S2. European Committee on Antimicrobial Susceptibility Testing breakpoints for *Enterobacterales*

Antimicrobial	Minimum inhibitory concentration (µg/mL)	
	Susceptible	Resistant
Ampicillin	≤8	>8
Amoxicillin	≤8	>8
Amoxicillin/clavulanic acid	≤8	>8
Cefalexin	≤16	>16
Gentamicin	≤2	>2
Norfloxacin	≤0.5	>0.5
Trimethoprim/sulfamethoxazole	≤2	>4

Supplementary Table S3. European Committee on Antimicrobial Susceptibility Testing tentative epidemiological cut-off values

Antimicrobial	Minimum inhibitory concentration (µg/mL)					
	Citrobacter freundii	*Enterobacter cloacae*	*Enterobacter gergoviae /hormaechei /ludwigii*	*E. coli*	*Serratia odorifera*	*Serratia marcescens*
Ampicillin	-	-	-	8	-	-
Amoxicillin/clavulanic acid	-	-	-	8	-	-
Cefalexin	-	-	-	32	-	-
Cephalotin	-	-	-	32	-	-
Doxycycline	8	8	-	8	-	-
Enrofloxacin	-	-	-	0.125	-	-
Florfenicol	-	-	-	16	-	-
Flumequine	-	-	-	2	-	-
Gentamicin	2	2	-	2	-	2
Neomycin	-	-	-	8	-	-
Streptomycin	-	-	-	16	-	-
Tetracycline	-	8	-	8	-	-
Trimethoprim/sulfamethoxazole	-	0.5	-	0.5	-	2

Supplementary Table S4. Clinical and Laboratory Standards Institute M45 Methods for Antimicrobial Dilution and Disk Susceptibility Testing of Infrequently Isolated or Fastidious Bacteria breakpoints for *Bacillus* spp. except *B. anthracis*

Antimicrobial	Minimum inhibitory concentration (µg/mL)		
	Susceptible	Intermediate	Resistant
Ampicillin	≤0.25	-	≥0.5
Clindamycin	≤0.25	1–2	≥4
Erythromycin	≤0.5	1–4	≥8
Gentamicin	≤0.5	8	≥16
Penicillin	≤0.12	-	≥0.25
Tetracycline	≤4	8	≥16
Trimethoprim/sulfamethoxazole	≤2/38	-	≥4/76

Supplementary Table S5. European Committee on Antimicrobial Susceptibility Testing breakpoints for *Bacillus* spp. except *B. anthracis*

Antimicrobial	Minimum inhibitory concentration (µg/mL)	
	Susceptible	Resistant
Clindamycin	≤1	>1
Erythromycin	≤0.5	>0.5

Supplementary Table S6. Clinical and Laboratory Standards Institute VET06 Methods for Antimicrobial Susceptibility Testing of Infrequently Isolated or Fastidious Bacteria Isolated From Animals breakpoints for *Moraxella* spp.

Antimicrobial	Minimum inhibitory concentration (µg/mL)		
	Susceptible	Intermediate	Resistant
Ampicillin	≤0.25	-	≥0.5
Erythromycin	≤2	-	*
Florfenicol	≤2	4	≥8
Penicillin	≤0.25	-	≥0.5
Tetracycline	≤2	4	≥8

* – for some organism/antimicrobial agent combinations, the absence or rare occurrence of resistant strains precludes defining any result categories other than "susceptible"

Supplementary Table S7. Clinical and Laboratory Standards Institute M45 Methods for Antimicrobial Dilution and Disk Susceptibility Testing of Infrequently Isolated or Fastidious Bacteria breakpoints for *Leuconostoc* spp.

Antimicrobial	Minimum inhibitory concentration (µg/mL)		
	Susceptible	Intermediate	Resistant
Ampicillin	≤8	-	*
Penicillin	≤8	-	*

* – for some organism/antimicrobial agent combinations, the absence or rare occurrence of resistant strains precludes defining any result categories other than "susceptible"

Supplementary Table S8. Clinical and Laboratory Standards Institute (CLSI) VET06 Methods for Antimicrobial Susceptibility Testing of Infrequently Isolated or Fastidious Bacteria Isolated From Animals and CLSI M45 Methods for Antimicrobial Dilution and Disk Susceptibility Testing of Infrequently Isolated or Fastidious Bacteria breakpoints for *Corynebacterium* spp. including *Corynebacterium diphtheriae* and related Coryneform genera

Antimicrobial	Minimum inhibitory concentration (µg/mL)		
	Susceptible	Intermediate	Resistant
Clindamycin	≤0.5	1	≥2
Doxycycline	≤4	8	≥16
Erythromycin	≤0.5	1	≥2
Gentamicin	≤4	8	≥16
Penicillin	≤0.12	0.25–2	≥4
Tetracycline	≤4	8	≥16
Trimethoprim/sulfamethoxazole	≤2/38	-	≥4/76

Supplementary Table S9. European Committee on Antimicrobial Susceptibility Testing breakpoints for *Corynebacterium* spp. other than *C. diphtheriae* and *C. ulcerans*

Antimicrobial	Minimum inhibitory concentration (µg/mL)	
	Susceptible	Resistant
Benzylpenicillin	≤0.001	>1
Clindamycin	≤0.5	>0.5
Tetracycline	≤2	>2

Supplementary Table S10. European Committee on Antimicrobial Susceptibility Testing breakpoints for *Corynebacterium diphtheriae*

Antimicrobial	Minimum inhibitory concentration (µg/mL)	
	Susceptible	Resistant
Amoxicillin	≤1	>1
Benzylpenicillin	≤0.001	>1
Clindamycin	≤0.5	>0.5
Doxycycline	≤0.5	>0.5
Erythromycin	≤0.06	>0.06
Tetracycline	≤1	>1
Trimethoprim/sulfamethoxazole	≤0.5	>0.5

Supplementary Table S11. Clinical and Laboratory Standards Institute M100 Ed31 Performance Standards for Antimicrobial Susceptibility Testing and European Committee on Antimicrobial Susceptibility Testing breakpoints for *Stenotrophomonas maltophilia*

Antimicrobial	Minimum inhibitory concentration (µg/mL)	
	Susceptible	Resistant
CLSI Trimethoprim/sulfamethoxazole	≤2/38	≥4/76
EUCAST Trimethoprim/sulfamethoxazole	≤0.001	>4

Supplementary Table S12. Tentative epidemiological cut-off values set by the European Committee on Antimicrobial Susceptibility Testing

Antimicrobial	Minimum inhibitory concentration (µg/mL)	
	Corynebacterium diphtheriae	*Stenotrophomonas maltophilia*
Benzylpenicillin	1	-
Clindamycin	0.5	-
Doxycycline	0.25	-
Erythromycin	0.016	-
Tetracycline	-	64
Trimethoprim/sulfamethoxazole	-	2
